# Integrated Analysis of Key Genes and Pathways Involved in Nonalcoholic Steatohepatitis Improvement After Roux-en-Y Gastric Bypass Surgery

**DOI:** 10.3389/fendo.2020.611213

**Published:** 2021-02-02

**Authors:** Fu Chen, Yong Zhou, Zhiyuan Wu, Yunze Li, Wenlong Zhou, Yong Wang

**Affiliations:** ^1^ Department of General Surgery, Fourth Affiliated Hospital of China Medical University, Shenyang, China; ^2^ Department of Colorectal and Hernia Minimally Invasive Surgery, Shengjing Hospital of China Medical University, Shenyang, China; ^3^ Department of General Surgery, The Third Hospital of Shenyang Medical College, Shenyang, China

**Keywords:** bariatric surgery, gastric bypass, nonalcoholic steatohepatitis, nonalcoholic fatty liver disease, microarray, differentially expressed genes

## Abstract

**Background:**

As the incidence of nonalcoholic fatty liver disease (NAFLD) increases globally, nonalcoholic steatohepatitis (NASH) has become the second common cause of liver transplantation for liver diseases. Recent evidence shows that Roux-en-Y gastric bypass (RYGB) surgery obviously alleviates NASH. However, the mechanism underlying RYGB induced NASH improvement is still elusive.

**Methods:**

We obtained datasets, including hepatic gene expression data and histologic NASH status, at baseline and 1 year after RYGB surgery. Differentially expressed genes (DEGs) were identified comparing gene expression before and after RYGB surgery in each dataset. Common DEGs were obtained between both datasets and further subjected to functional and pathway enrichment analysis. Protein–protein interaction (PPI) network was constructed, and key modules and hub genes were also identified.

**Results:**

In the present study, GSE106737 and GSE83452 datasets were included. One hundred thirty common DEGs (29 up-regulated and 101 down-regulated) were identified between GSE106737 and GSE83452 datasets. KEGG analysis showed that mineral absorption, IL-17 signaling pathway, osteoclast differentiation, and TNF signaling pathway were significantly enriched. Based on the PPI network, *IGF1*, *JUN*, *FOS*, *LDLR*, *TYROBP*, *DUSP1*, *CXCR4*, *ATF3*, *CXCL2*, *EGR1*, *SAA1*, *CTSS*, and *PPARA* were identified as hub genes, and three functional modules were also extracted.

**Conclusion:**

This study identifies the global gene expression change in the liver of NASH patients before and after RYGB surgery in a bioinformatic method. Our findings will contribute to the understanding of molecular biological changes underlying NASH improvement after RYGB surgery.

## Introduction

In the past few decades, obesity and its comorbidities are becoming the leading causes of death around the world (1). Meanwhile, nonalcoholic fatty liver disease (NAFLD) has become the most prevalent chronic liver disease in the United States (2). NAFLD consists of a spectrum of pathological states ranging from simple steatosis to nonalcoholic steatohepatitis (NASH) (2). Epidemiological studies show that 59.1% of biopsy-proven NAFLD patients progress to NASH (2, 3). As the incidence of advanced liver diseases such as cirrhosis and hepatocarcinoma are significantly increased in NASH patients, NASH becomes the second common cause of liver transplantation for liver diseases and is still growing (2).

So far, bariatric surgery is the most effective approach to treat obesity, which can also alleviate its comorbidities, such as type II diabetes mellitus (T2DM), hypertension, and hyperlipidemia (4). Roux-en-Y gastric bypass (RYGB) and sleeve gastrectomy (SG) serve as the most prevalent bariatric procedures in the world (5). Now, RYGB is recognized as the gold standard bariatric procedure worldwide (6). Recently, a study showed that all NAFLD parameters improved after bariatric surgery. This effect is more significant in patients undergoing RYGB surgery than patients who undergo adjustable gastric banding (AGB) surgery (7). Evidence from the liver biopsy showed that hepatic steatosis, hepatocellular ballooning, lobular inflammation, fibrosis, and NAFLD score obviously improved 1 year after bariatric surgery (8). The remission rate of NASH is over 85% after bariatric surgery (8). However, the mechanism of bariatric surgery in NASH alleviation is still elusive.

RYGB surgery is thought to be the malabsorptive procedure that bypasses a great portion of the intestine leading to nutrient malabsorption and weight loss (9). In the past few decades, evidence shows that bariatric surgery contributes to weight loss at least partially through energy balance regulation, peripheral and central nervous system regulation, gastrointestinal absorption and secretion, and microbiota alteration (10). Although the therapeutic effect of bariatric surgery on NASH has been confirmed recently, the underlying mechanism is almost unknown.

Microarray technology is an essential tool to illustrate gene expression patterns in multiple diseases, which can help us understand the biology and molecular mechanisms of diseases more efficiently. Microarray technology has also been used in the liver of NASH patients and animal models to detect differentially expressed genes (DEGs), screen disease biomarkers, or find new therapeutic targets (11, 12). By searching the Gene Expression Omnibus (GEO), there exists only two human datasets comparing DEGs in the liver tissue of NASH patients before and after bariatric surgery (13, 14). As the bioinformation in these datasets have not been thoroughly mined. Thus, we performed a global transcriptome analysis using a bioinformatic approach to find pivotal genes which might mediate RYGB surgery induced NASH improvement. In addition, hub genes and functional modules were identified in the DEGs, and biological function and pathway annotation were also performed.

## Material and Methods

### Microarray Datasets Collection

The gene expression datasets were obtained from the Gene Expression Omnibus (GEO) database (http://www.ncbi.nlm.nih.gov/geo). Datasets must meet the main inclusion criteria: 1) Datasets assessed transcriptome information in the liver specimen of patients who undergone RYGB surgery; 2) Datasets included liver specimens during RYGB surgery and paired liver specimens collected through liver biopsy 1-year post-operation; 3) All cases should be pathological proven NASH before RYGB surgery and NASH regression 1 year post-operation; 4) Datasets must contain at least 10 cases and paired follow-ups. In the GSE106737 dataset, 21 cases matched the inclusion criteria. In the GSE83452 dataset, 16 cases matched the inclusion criteria. All microarray analysis in the GSE106737 and the GSE83452 were performed on the GPL16686 platform. All gene expression patterns were originated from the open-access GEO database, so our study did not require Ethics Committee approval.

### Datasets Analysis

The Gene expression matrix and attached annotation document for GSE106737 and GSE83452 datasets were downloaded from the GEO database. GSE files were divided into Baseline (patients with pathological proven NASH during RYGB surgery) and Follow-up (same patients with NASH improvement 1 year after RYGB surgery) groups. All microarray data had already been corrected and normalized by the RMA method. Gene IDs in the matrix were annotated with gene symbols by R package through related annotation documents. Mean values were preserved if the gene symbols matched with multiple probes. The DEGs were detected between liver biopsies at baseline and 1 year after RYGB surgery in each microarray by limma (linear models for microarray) R package. In each dataset, |log fold change (FC)| > 0.5 and P-value < 0.05 were regarded as the threshold value to determine DEGs. Then, DEGs in each dataset were upload to Venn software online (http://bioinformatics.psb.ugent.be/webtools/Venn/) to get the common DEGs.

### GO and KEGG Enrichment Analysis

Gene Ontology (GO) is a recognized method to annotate the function of genes detected through high throughput transcriptomic or genomic data. Kyoto Encyclopedia of Genes and Genomes (KEGG) is a database resource including genomes, diseases, biological pathways, and other bioinformation. In this study, Bioconductor clusterProfiler package was used to carried out GO and KEGG analysis for common DEGs (15). Biological process (BP), cellular component (CC), molecular function (MF), and pathways that were significantly enriched were screened out when the *P*-value < 0.05.

### PPI Network Construction and Module Analysis

The Search Tool for the Retrieval of Interacting Genes/Proteins (STRING, https://string-db.org/) (version 11) is a frequently used software online to predict the interaction between proteins and proteins (16). In this study, the PPI network was predicted by STRING online database setting the cut-off value 0.04. Then, the PPI networks were visualized and analyzed with Cytoscape software (version 3.8.0). Hub genes were identified using CytoHubba plug-in APP in Cytoscape with the cut-off value degree value >10. Vital modules in the PPI network were clustered using the Molecular Complex Detection (MCODE) plug-in APP in Cytoscape (17). The cut-off value was MCODE score ≥ 5.

## Results

### Information of Selected Cases

Initially, GSE106737 and GSE83452 were included according to the inclusion criteria. However, to obtain high-quality data, we further screen both datasets to get cases that match inclusion criteria (details described in “*Material and Methods*”). There existed 37 Baseline (patients with pathological proven NASH during RYGB surgery) and 37 paired Follow-up (same patients with NASH improvement 1 year after RYGB surgery) for further analysis. The detailed information of included cases was shown in **Table 1** and **Supplementary Tables 1** and **2**.

**Table 1:** Characteristics of the included microarray datasets.

GSE ID	Baseline	Follow-up(1 year)	Tissues	Analysis Type	Platform	Year
GSE106737	NASH (21 cases)	NASH improvement (21 paired cases)	Liver	Array	GPL16686	2017
GSE83452	NASH (16 cases)	NASH improvement (16 paired cases)	Liver	Array	GPL16686	2016

### Identification of DEGs Between Baseline and Follow-Up

**Figure 1:**
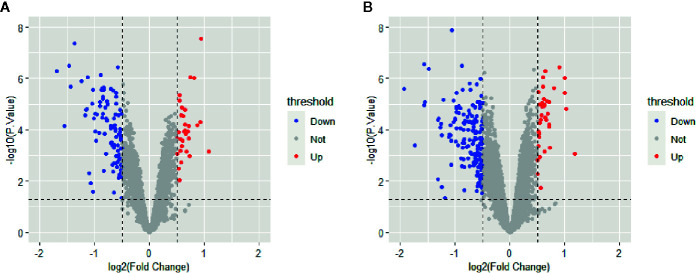
Volcano plots of gene expression in GSE106737 **(A)** and GSE83452 **(B)**, with the threshold of P<0.05 and |log FC| > 0.5. Blue points represented down-regulated genes, red points represented up-regulated genes, and gray points represented genes with on significant difference.

**Figure 2:**
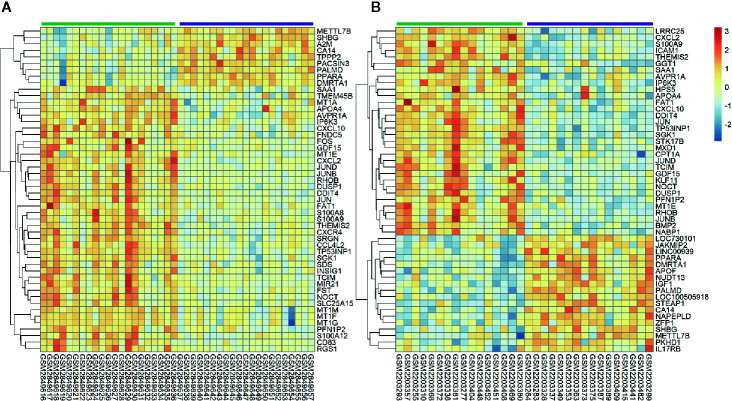
Heatmaps of top 50 genes expression in GSE106737 **(A)** and GSE83452 **(B)**. Blue square represented down-regulated genes and red square represented up-regulated genes. The green bar represented baseline gene expression and the purple bar represented follow-up gene expression.

All selected cases in GSE106737 and GSE83452 datasets were standardized to eliminate individual differences. Each dataset was homogeneous after standardization. We screened out 132 DEGs in GSE106737. Among these DEGs, 31 genes were up-regulated, and 101 genes were down-regulated. In GSE83452, we identified 206 DEGs in which 38 genes were up-regulated, and 168 genes were down-regulated. The volcano plots and heatmaps of both GSE106737 and GSE83452 were shown in **Figures 1** and **2**, respectively.

### Identification of Common DEGs Between GSE106737 and GSE83452

**Figure 3:**
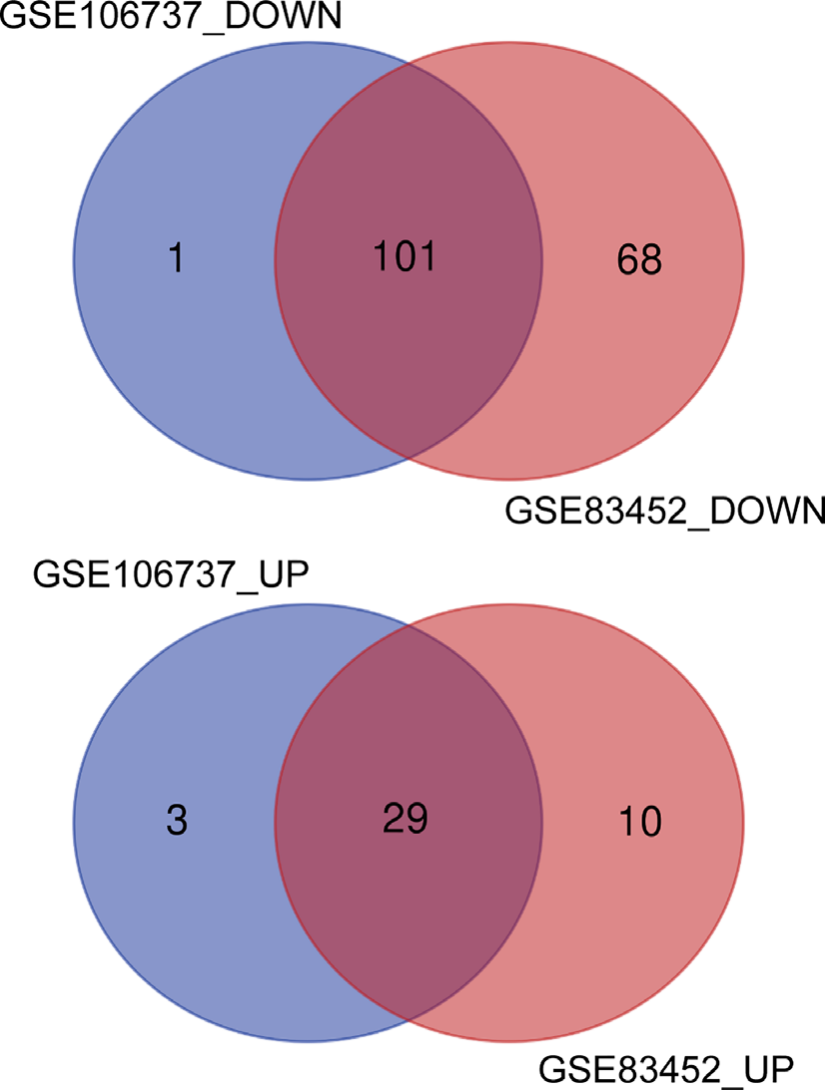
Identification of common DEGs from GSE106737 and GSE83452 using the Venn diagram. The cross section represented the common DEGs. DOWN, down-regulated DEGs; UP, up-regulated DEGs.

DEGs in both GSE106737 and GSE83452 datasets were analyzed using the Venn diagram. We obtain 130 common DEGs between two datasets, in which 29 DEGs were up-regulated (logFC > 0) and 101 DEGs were down-regulated (logFC < 0). Venn diagram was shown in **Figure 3**, and the details of common DEGs were shown in **Table 2**.

**Table 2:** 130 common DEGs in NASH patients with paired liver specimens at baseline and 1-year post-operation.

Regulation	Gene Symbol
Down	*IGFBP1*, *MT1G*,*VSIG4*, *MT1B*, *FOS*, *OSBPL11*, *CXCL10*, *LPIN2*, *FNDC5*, *JUNB*, *SDS*, *PLIN2*, *TP53INP1*, *RND3*, *VTRNA1-1*, *CXCR4*, *UPP2*, *FGL2*, *RHOB*, *ETNPPL*, *S100A12*, *SRGN*, *LINC-PINT*, *FOSL2*, *PEG10*, *GDF15*, *KLF10*, *ASCL1*, *MT1F*, *CTSS*, *JUND*, *MFSD2A*, *TMEM45B*, *TYROBP*, *CLGN*, *NOCT*, *AVPR1A*, *LURAP1L*, *FST*, *CXCL9*, *TIMD4*, *THBS1*, *FPR3*, *LDLR*, *JUN*, *TSPAN3*, *CXCL16*, *SGK1*, *SDC4*, *NRG1*, *ZBTB16*, *PFN1P2*, *S100A8*, *FADS1*, *ARRDC4*, *DDIT4*, *NAMPT*, *FAT1*, *GPNMB*, *LAPTM5*, *MT1E*, *TCIM*, *THEMIS2*, *FCN3*, *CD53*, *SIK1*, *TXNIP*, *CCL4L2*, *NNMT*, *MT1M*, *INSIG1*, *ATF3*, *ACSL4*, *DUSP1*, *SQLE*, *PRAMEF10*, *EGR1*, *CXCL2*, *LRRC31*, *CD68*, *SAA1*, *FOSB*, *MNDA*, *MT1L*, *RGS1*, *CD83*, *SLC25A15*, *NCAM2*, *S100A9*, *FGF21*, *MT1H*, *PDE11A*, *IP6K3*, *KLF6*, *BIRC3*, *APOA4*, *GABARAPL1*, *SYBU*, *MT1A*, *MIR21*
Up	*IL17RB*, *DMRTA1*, *SHBG*, *NAPEPLD*, *NECAB2*, *APOF*, *CA14*, *A2M*, *MOGAT1*, *RPL19P12*, *PKHD1*, *PPARA*, *IGF1*, *FOLH1*, *JAKMIP2*, *PALMD*, *HNF1A-AS1*, *PCDH18*, *METTL7B*, *STEAP1*, *PACSIN3*, *TPPP2*, *TBX3*, *CMYA5*, *LOC730101*, *LOC100505918*, *LINC00939*, *MIR192*

### GO and KEGG Enrichment Analysis

**Figure 4:**
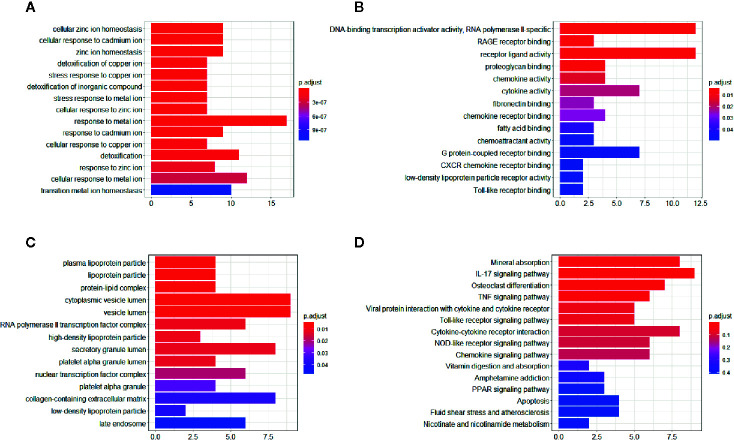
GO and KEGG enrichment analysis of the common DEGs. **(A)** Significant enriched biological process (BP) in GO terms. **(B)** Significant enriched cell component (CC) in GO terms. **(C)** Significant enriched molecular function (MF) in GO terms. **(D)** Significant enriched KEGG pathway in common DEGs.

All common DEGs were analyzed by the clusterProfiler package to illustrate the biological functions and pathways related to DEGs. The results demonstrated that cellular zinc ion homeostasis process (*P*-value= 5.97E-13) was the most significantly enriched biological process (BP), followed by cellular response to cadmium ion process (*P*-value= 7.84E-13), zinc ion homeostasis process (*P*-value= 1.02E-12), and so on. Cell component (CC) and metabolic function (MF) of DEGs were mainly related to lipoprotein homeostasis and inflammatory signaling pathway, respectively. The detailed information of GO analysis was shown in **Figure 4**. KEGG pathway analysis indicated that DEGs are mainly related to mineral absorption pathway (*P*-value= 3.75E-08), followed by IL-17 signaling pathway (*P*-value= 1.11E-07), osteoclast differentiation (*P*-value= 1.23E-04), etc. The top 10 pathways of KEGG analysis were shown in **Figure 4**.

### PPI Network Construction and Hub Genes Identification

**Figure 5:**
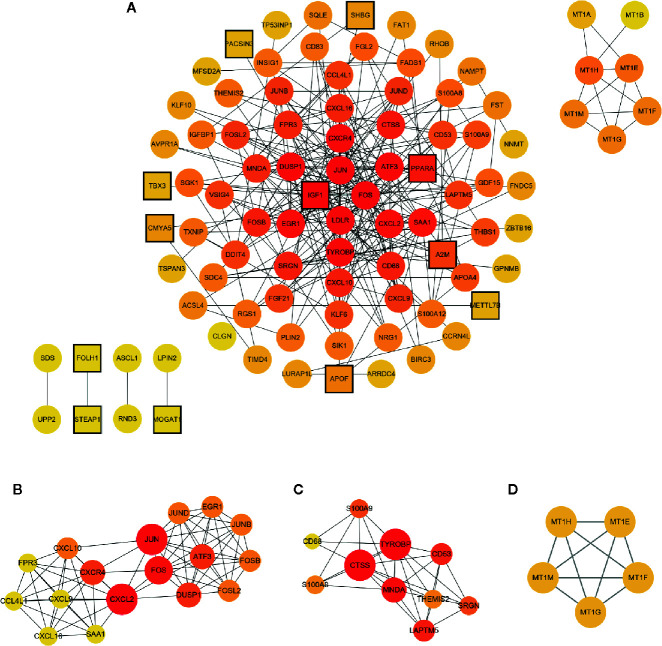
PPI network of the common DEGs and module exhibition. **(A)** The PPI network consisted of 93 genes. **(B–D)** Modules 1–3 which were extracted from the PPI network. The circle represented down-regulated genes, while the square with black border represented up-regulated genes. The depth of color correlated with the number of connections between nodes. In **(B–D)**, the size of the circle correlated with the number of connections between nodes in each module.

The PPI network of the common DEGs was constructed using the STRING online database and further analyzed and visualized using Cytoscape software. In the PPI network, there existed 93nodes, including 12 up-regulated genes and 81 down-regulated genes. Among 130 common DEGs, 37 genes including 17 up-regulated genes and 20 down-regulated genes were excluded from the PPI network. The term “degree” calculated using CytoHubba plug-in APP in Cytoscape indicated the number of interactions between genes or nodes. The PPI network was shown in **Figure 5**, the color depth positively correlated with the degree value. Setting the cut-off value of degree>10, *IGF1*, *JUN*, *FOS*, *LDLR*, *ATF3*, *TYROBP*, *DUSP1*, *CXCR4*, *CXCL2*, *EGR1*, *SAA1*, *PPARA*, and *CTSS* were identified as the hub genes. Among 13 hub genes, IGF1 and PPARA were up-regulated, and other genes were down-regulated.

### Module Analysis of the PPI Network

Modules were extracted using MCODE plug-in APP in Cytoscape. Setting the threshold MCODE score ≥ 5, we obtained 3 modules from the PPI network, including Module 1 (MCODE Score=8.875, Nodes=17), Module 2 (MCODE Score=6.444, Nodes=10), and Module 3 (MCODE Score=5, Nodes=5). All genes belonged to these three modules were down-regulated common DEGs. Modules were shown in **Figure 5**. The genes in each module were analyzed using the clusterProfiler package to identify their GO and KEGG enrichment. Module 1 was mainly enriched in the IL-17 signaling pathway, osteoclast differentiation pathway, and chemokine signaling pathway. Module 2 was mainly involved in the lysosome pathway, IL-17 signaling pathway, and antigen processing and presentation pathway. Module 3 take part in the mineral absorption pathway. The detailed information was shown in **Table 3**.

**Table 3:** Characteristic of each module and GO and KEGG pathway analysis of genes in each module.

Cluster	Score	Nodes	Edges	Regulation	Gene Symbol	Gene Ontology (Top 5)	KEGG Pathway
1	8.875	17	71	Down	*CXCL9*, *CXCL10*, *SAA1*, *CXCL2*, *FOS*, *JUND*, *CCL4L1*, *JUN*, *JUNB*, *CXCL16*, *ATF3*, *CXCR4*, *FPR3*, *FOSL2*, *DUSP1*, *FOSB*, *EGR1*	· Leukocyte chemotaxis · Cell chemotaxis · Response to cAMP · Response to chemokine · Cellular response to chemokine	· IL-17 signaling pathway · Osteoclast differentiation · Chemokine signaling pathway · Viral protein interaction with cytokine and cytokine receptor · Toll-like receptor signaling pathway…
2	6.444	10	29	Down	*TYROBP*, *CD68*, *THEMIS2*, *MNDA*, *S100A9*, *S100A8*, *LAPTM5*, *CD53*, SRGN, CTSS	· Neutrophil degranulation · Neutrophil activation involved in immune response · Neutrophil activation · Neutrophil mediated immunity · Activation of innate immune response	· Lysosome · IL-17 signaling pathway · Antigen processing and presentation
3	5.000	5	10	Down	*MT1F*, *MT1E*, *MT1G*, *MT1M*, *MT1H*	· Detoxification of copper ion · Stress response to copper ion · Detoxification of inorganic compound · Stress response to metal ion · Cellular response to zinc ion	· Mineral absorption

## Discussion

In the past few decades, NAFLD has become the most common chronic liver disease around the world (18, 19). Although NAFLD is a benign disease, NASH (a progressive stage of NAFLD) is the second common cause for liver transplantation and increases hepatocarcinoma progression (2). So far, bariatric surgery seems to be the most long-lasting effective method to treat NASH (7, 8). However, due to the safety issue, the American Association for the Study of Liver Diseases has not recommended bariatric surgery to specifically treat NASH (20). However, it can serve as an option for obese patients (BMI ≥35 kg/m2) with one or more complications remediable by weight loss, including NAFLD and NASH (21). Nowadays, less invasive and safer modern interventions are needed to replace invasive bariatric procedures. So, it is urgently needed to illustrate the mechanism underlying bariatric surgery in NASH improvement. Microarray technology is an efficient method to get transcriptomic bioinformation under various diseases. In this study, we included two microarray studies comparing gene expression profiles between Baseline (liver specimens obtained during RYGB surgery) and Follow-up (liver specimens obtained 1 year after RYGB surgery) to get DEGs. Furthermore, we perform functional annotation and construct PPI network to illustrate the biological function and involved pathways of the DEGs.

Although the GSE106737 dataset had been already used by others, their research was focused on the function of CD8 T cells in the progression and remission process in NASH (14). The study using the GSE83452 dataset included a wide range of patients with NASH or fibrosis and focused on single gene interpretation (13). The present research strictly selected cases which verified NASH improvement after RYGB surgery to interpret the hepatic global transcriptomic change underlying this process. In the present study, we obtained 130 common DEGs between GSE106737 and GSE83452 datasets (cases selected strictly matched inclusion criteria). Among these DEGs, 29 genes were up-regulated, and 101 genes were down-regulated. PPI network included 93 genes and most of them belonged to the down-regulated DEGs. In the PPI network, *IGF1*, *JUN*, *FOS*, *LDLR*, *TYROBP*, *DUSP1*, *CXCR4*, *ATF3*, *CXCL2*, *EGR1*, *SAA1*, *CTSS*, and *PPARA* were identified as the hub genes and ranked by the degree value using CytoHubba plug-in APP in Cytoscape. Among 13 hub genes, only *IGF1* and *PPARA* were up-regulated, while others were down-regulated. In consistence with this conclusion, a previous study had demonstrated that PPARα activation might be the mechanism underlying NASH improvement after RYGB surgery (13, 22).

Furthermore, using the MCODE plug-in APP in Cytoscape, we got three modules in the PPI network. Seventeen genes were included in Module 1, among which, *JUN*, *FOS*, *DUSP1*, *CXCR4*, *ATF3*, *CXCL2*, *EGR1*, and *SAA1* severed as hub genes. These genes participated in the IL-17 signaling pathway, osteoclast differentiation pathway, chemokine signaling pathway, viral protein interaction with cytokine and cytokine receptor pathway, etc. Activator protein 1 (AP-1), which was combined with Jun (c-Jun, JunB, and JunD), Fos (c-Fos, FosB, Fra-1, and Fra-2), activating transcription factor (Atf) and musculoaponeurotic fibrosarcoma (Maf) proteins to form homodimer or heterodimer is a dimeric leucine zipper (bZIP) transcription factor (23). Generally, the AP-1 complex played a pivotal role in acute stress response in the liver (24). Recent evidence showed that c-Jun/AP-1 overexpression might mediate the hepatic pathological alteration in NASH patients (25). Furthermore, AP-1 correlated with hepatic lipid metabolism and NASH progression through regulating PPARγ expression (26). CXCL and CCL are chemokines with chemotactic properties and CXCR is one type of G protein-coupled chemokine receptors (27). Chemokines participate in homeostatic or inflammatory regulation which mediates physiological or pathophysiological alteration in disease progression through binding corresponding receptors (28). CXCL2 and CXCL8 chemokines were mainly originated from activated Kupffer cells (29). CXCL2 and CXCL8 with neutrophil chemotactic properties recruit neutrophils, releasing reactive oxygen species (ROS) and proteases and therefore initiating hepatocyte necrosis (30). CXCL9, CXCL10, and CXCL 16 attract lymphocytes or natural killer (NK) cells involved in hepatic inflammatory pathogenesis and accelerate hepatic fibrosis progression (31). CXCR4 is the corresponding receptor of CXCL12. In the liver of NASH patients, the affinity of CXCR4 significantly increased which increased CD4+ T-cells deposition (32). DUSPs, namely, dual-specificity protein tyrosine phosphatases served as mitogen-activated protein kinases (MAPK) inactivator (33). DUSPs as MAPK phosphatases (MKPs) dephosphorylated the threonine and tyrosine residues of MAPK and played a pivotal role in hepatic metabolic regulation (34). MKP-1, a negative regulator of p38 MAPK and c-Jun NH_2_-terminal kinase 1/2 (JNK1/2), activated transcription factors regulating hepatic lipid homeostasis (35). Studies showed that MKP-1 deficient protected mice from diet-induced obesity and diet or gene induced hepatic steatosis (35, 36).EGR1, namely, early growth response 1, played an essential role in the pathophysiological process of inflammation and tissue repairment (37). A previous bioinformatic study identified that the lower expression of hepatic EGR1 might promote NAFLD development (38). Evidence from both *in vivo* and *in vitro* experiments also confirmed the function of EGR1 in hepatic insulin response and hepatic lipid metabolic regulation (37). SAA1, namely, serum amyloid A-1 protein belongs to the SAA family (39). As a classical acute-phase protein produced by hepatocytes, SAA mediated infection, injury, and inflammation response and could regulate toll-like receptor 4 (TLR4), which participated in obesity-induced insulin resistance (40). In progressive liver diseases, including NASH, the serum level of SAA could serve as a biomarker for inflammatory status (41). Moreover, SAA1 had the ability to stimulate NF-κBp65 protein transportation from the cytoplasm to the nucleus and activate the NF-κB pathway which mediated NASH progression (40).

Module 2 included *TYROBP*, *CD68*, *THEMIS2*, *MNDA*, *S100A9*, *S100A8*, *LAPTM5*, *CD53*, *SRGN*, and *CTSS*, among which TYROBP and CTSS served as the hub genes in the PPI network. TYROBP, namely, Tyrosine kinase binding protein could activate NK cells through binding a variety of receptors (42). Studies showed that TYROBP could synthesize lipopolysaccharide and activated pro-inflammatory cytokines production (43). After TYROBP gene deletion, the pro-inflammatory cytokines decreased obviously, which indicated that TYROBP might be involved in NASH progression (44). In Module 2, *CTSS*, *LAPTM5*, and *CD68* were involved in the lysosome pathway. CTSS and CD68 were both recognized as the molecular biomarker of macrophages (45). Moreover, CTSS and CD68 positively correlated with hepatic macrophage infiltration in NAFLD mice (45). Meanwhile, a recent bioinformatic study identified hepatic CTSS and CD68 as major genes contributing to NAFLD (46). Furthermore, we identified *S100A9* and *S100A8* genes in Module 2, which were involved in the IL-17 signaling pathway. As members of the S100 proteins, S100A8 and S100A9 could be secreted by either neutrophils or monocytes (47). S100A8, S100A9, and S100A8/S100A9 (heterodimer formed by S100A8 and S100A9) were implicated in various inflammatory diseases and could serve as the biomarker for inflammatory activity monitoring (48). It was worth noting that S100A8 and S100A9 had a direct link with inflammatory and fibrotic status in NASH patients (47).

Module 3 included *MT1E*, *MT1F*, *MT1G*, *MT1H*, and *MT1M*. Though none of them was identified as the hub gene, they were involved in the mineral absorption pathway. Metallothioneins (MTs), including MT1, MT2, MT3, and MT4 isoforms, played a pivotal role in heavy metal toxicity protection, metal homeostasis, and oxidative stress regulation (49). In MT1, there existed MT1A, MT1B, MT1E, MT1F, MT1G, MT1H, MT1M, and MT1X isoforms (49). These proteins mainly regulated copper and zinc homeostasis in the liver and protected hepatocytes from oxidative damage (50). Thus, MT1 expression negatively correlated with the damage status of chronic liver diseases (51). Moreover, MT1 could stimulate damaged hepatocyte repair and regeneration (52). However, we could not ignore the fact that mineral deficiency was a common complication after RYGB surgery. Data from the American Society for Metabolic and Bariatric Surgery (ASMBS) Integrated Health Nutritional Guidelines showed that the prevalence of zinc deficiency occurred in 40% post-RYGB patients and copper deficiency occurred in 10%–20% post-RYGB patients (53). Thus, we speculated that copper and zinc deficiency might be caused by the low hepatic MT1 expression after RYGB surgery. On the contrary, as MT1 protein expression was positively regulated by zinc and copper, the zinc and copper deficiency further suppressed MT1 expression and formed a vicious circle (54, 55). So, the mineral condition should be inspected strictly and supplements should be administrated if necessary after RYGB surgery.

Though we got much transcriptomic bioinformation underlying RYGB induced NASH improvement, the causes of these hepatic changes had not been fully clarified. Due to the lack of conclusive evidence, we speculated about the reason for inflammatory related genes alteration after RYGB surgery: 1) The gastrointestinal anatomy was altered after RYGB surgery, so more bile acid (BA) reach ileum and stimulate enteric GLP-1 and PYY release through activating the local L-cells (56). Furthermore, the increased BA also stimulated TGR5 expression in Kupffer cells (57). These BA induced metabolic change may lead to a reduction in hepatic pro-inflammatory genes expression after RYGB surgery. 2) RYGB surgery manipulate a variety of adipocyte derived adipocytokines (adiponectin, leptin, TNFα, IL-6, and etc.), which have been proven to be involved in the hepatic inflammatory process (58). For instance, RYGB surgery could significantly increase adiponectin levels, which suppress Kupffer cells and hepatic stellate cells (HSC) activation and decrease hepatic inflammatory genes expression (58, 59). 3) The gut microbiota composition obviously shifted after RYGB surgery, as the rearrangement of the gastrointestinal tract created a more acidic and oxygen-rich environment (60). Recent evidence showed that specific gut microbiota was independently associated with the severity of NAFLD (61). We speculated that gut microbiota shift after RYGB surgery might alleviate NASH through modulating hepatic inflammatory gene expression.

There existed several limitations of the present study, which should be noted. First and foremost, due to the difficulty in collecting the hepatic specimens after RYGB surgery, we did not validate the genes in the pathway highlighted by the present study. This limited the strength of our results in interpreting the mechanism underlying RYGB surgery induced NASH alleviation. Second, we could not get enough demographic and clinical information for the patients included in our study. Considering body weight, body mass index (BMI), and obesity related comorbidities such as type 2 diabetes mellitus were all risk factors for NASH. There existed confounding factors in the analysis in the present study. Third, hepatic tissue was the heterogeneous mixture of hepatocytes and mesenchymal cells, and the cell composition might be influenced during specimen collection. Finally, the metabolic change in the liver after bariatric surgery is a dynamic process. Analysis at a single time point after surgery ignored the full picture of the hepatic remodeling process.

## Conclusion

In conclusion, in the present study, we exhibit the global profile of DEGs and corresponding signaling pathways, which may mediate RYGB surgery induced NASH improvement. In the process of RYGB surgery induced NASH improvement, the possible key genes are *IGF1*, *JUN*, *FOS*, *LDLR*, *TYROBP*, *DUSP1*, *CXCR4*, *ATF3*, *CXCL2*, *EGR1*, *SAA1*, *CTSS*, and *PPARA*, and the possible involved pathways are IL-17 signaling pathway, osteoclast differentiation pathway, chemokine signaling pathway, viral protein interaction with cytokine and cytokine receptor pathway, Toll-like receptor signaling pathway, TNF signaling pathway and mineral absorption pathway. Most DEGs and enriched signaling pathways are involved in the inflammatory response, immunoreaction, and lipid homeostatic regulation. These results provide pivotal transcriptome information underlying RYGB surgery induced NASH alleviation.

## Data Availability Statement

The datasets presented in this study can be found in online repositories. The names of the repository/repositories and accession number(s) can be found in the article/**Supplementary Material**.

## Ethics Statement

Ethical review and approval was not required for the study on human participants in accordance with the local legislation and institutional requirements.

## Author Contributions

All authors listed have made a substantial, direct, and intellectual contribution to the work and approved it for publication.

## Funding

This work was supported by the Support Program for Young and Middle-aged Scientific and Technological Innovation Talent (Grant No. RC200607); 2020 “Double First Class”: Support Plan for the Iconic Achievement of Clinical Medicine (Grant No. 3110200345).

## Conflict of Interest

The authors declare that the research was conducted in the absence of any commercial or financial relationships that could be construed as a potential conflict of interest.
