# Supplementary material for: Integrated Analysis of Key Genes and Pathways Involved in Nonalcoholic Steatohepatitis Improvement After Roux-en-Y Gastric Bypass Surgery
Source: Front Endocrinol (Lausanne). 2021 Feb 2;11:611213. doi: 10.3389/fendo.2020.611213 (PMC7884850; doi:10.3389/fendo.2020.611213)
Supplement: Supplementary file 1 [file Table_1.docx]

**Supplementary Table 1** Selected 21 in 110 cases from GSE106737

| **Case** | **Baseline** | **Follow-up (Paired)** |
| --- | --- | --- |
| 1 | GSM2849616 | GSM2849637 |
| 2 | GSM2849617 | GSM2849638 |
| 3 | GSM2849618 | GSM2849639 |
| 4 | GSM2849619 | GSM2849640 |
| 5 | GSM2849620 | GSM2849641 |
| 6 | GSM2849621 | GSM2849642 |
| 7 | GSM2849622 | GSM2849643 |
| 8 | GSM2849623 | GSM2849644 |
| 9 | GSM2849624 | GSM2849645 |
| 10 | GSM2849625 | GSM2849646 |
| 11 | GSM2849626 | GSM2849647 |
| 12 | GSM2849627 | GSM2849648 |
| 13 | GSM2849628 | GSM2849649 |
| 14 | GSM2849629 | GSM2849650 |
| 15 | GSM2849630 | GSM2849651 |
| 16 | GSM2849631 | GSM2849652 |
| 17 | GSM2849632 | GSM2849653 |
| 18 | GSM2849633 | GSM2849654 |
| 19 | GSM2849634 | GSM2849655 |
| 20 | GSM2849635 | GSM2849656 |
| 21 | GSM2849636 | GSM2849657 |
