# Supplementary material for: Integrated Analysis of Key Genes and Pathways Involved in Nonalcoholic Steatohepatitis Improvement After Roux-en-Y Gastric Bypass Surgery
Source: Front Endocrinol (Lausanne). 2021 Feb 2;11:611213. doi: 10.3389/fendo.2020.611213 (PMC7884850; doi:10.3389/fendo.2020.611213)
Supplement: Supplementary file 2 [file Table_2.docx]

**Supplementary Table 2** Selected 16 in 231 cases from GSE83452

| **Case** | **Baseline** | **Follow-up (Paired)** |
| --- | --- | --- |
| 1 | GSM2203293 | GSM2203264 |
| 2 | GSM2203357 | GSM2203283 |
| 3 | GSM2203255 | GSM2203328 |
| 4 | GSM2203310 | GSM2203337 |
| 5 | GSM2203368 | GSM2203349 |
| 6 | GSM2203372 | GSM2203353 |
| 7 | GSM2203338 | GSM2203363 |
| 8 | GSM2203381 | GSM2203373 |
| 9 | GSM2203277 | GSM2203375 |
| 10 | GSM2203404 | GSM2203387 |
| 11 | GSM2203448 | GSM2203389 |
| 12 | GSM2203452 | GSM2203409 |
| 13 | GSM2203451 | GSM2203415 |
| 14 | GSM2203408 | GSM2203441 |
| 15 | GSM2203469 | GSM2203482 |
| 16 | GSM2203380 | GSM2203299 |
